# Inactivation of the Huntington's disease gene (*Hdh*) impairs anterior streak formation and early patterning of the mouse embryo

**DOI:** 10.1186/1471-213X-5-17

**Published:** 2005-08-18

**Authors:** Juliana M Woda, Teresa Calzonetti, Paige Hilditch-Maguire, Mabel P Duyao, Ronald A Conlon, Marcy E MacDonald

**Affiliations:** 1 Molecular Neurogenetics Unit, Center for Human Genetic Research, Massachusetts General Hospital, CNY-149, 13th Street, Charlestown MA 02129, USA; 2 Department of Genetics, Case Western Reserve University, 10900 Euclid Avenue, Cleveland, OH 44106, USA; 3 University of Queensland, Faculty of Health Sciences, St Lucia QLD 4072, Australia; 4 Department of Pathology, Harvard Medical School, 77 Avenue Louis Pasteur, NRB-850A, Boston MA 02115, USA

## Abstract

**Background:**

Huntingtin, the *HD *gene encoded protein mutated by polyglutamine expansion in Huntington's disease, is required in extraembryonic tissues for proper gastrulation, implicating its activities in nutrition or patterning of the developing embryo. To test these possibilities, we have used whole mount *in situ *hybridization to examine embryonic patterning and morphogenesis in homozygous *Hdh*^*ex*4/5 ^huntingtin deficient embryos.

**Results:**

In the absence of huntingtin, expression of nutritive genes appears normal but E7.0–7.5 embryos exhibit a unique combination of patterning defects. Notable are a shortened primitive streak, absence of a proper node and diminished production of anterior streak derivatives. Reduced *Wnt3a*, *Tbx6 *and *Dll1 *expression signify decreased paraxial mesoderm and reduced *Otx2 *expression and lack of headfolds denote a failure of head development. In addition, genes initially broadly expressed are not properly restricted to the posterior, as evidenced by the ectopic expression of *Nodal*, *Fgf8 *and *Gsc *in the epiblast and *T *(*Brachyury*) and *Evx1 *in proximal mesoderm derivatives. Despite impaired posterior restriction and anterior streak deficits, overall anterior/posterior polarity is established. A single primitive streak forms and marker expression shows that the anterior epiblast and anterior visceral endoderm (AVE) are specified.

**Conclusion:**

Huntingtin is essential in the early patterning of the embryo for formation of the anterior region of the primitive streak, and for down-regulation of a subset of dynamic growth and transcription factor genes. These findings provide fundamental starting points for identifying the novel cellular and molecular activities of huntingtin in the extraembryonic tissues that govern normal anterior streak development. This knowledge may prove to be important for understanding the mechanism by which the dominant polyglutamine expansion in huntingtin determines the loss of neurons in Huntington's disease.

## Background

Huntington's disease (HD) is a dominantly inherited neurodegenerative disorder that is caused by CAG repeats in the *HD *locus that extend a polyglutamine tract in a ubiquitous HEAT domain protein called huntingtin [1]. The molecular mechanism by which the new property that is conferred on huntingtin by the polyglutamine expansion leads to the hallmark loss of striatal neurons in HD is not known. However, polyglutamine expansions in unrelated proteins that target distinct neuronal cell populations cause distinct 'polyglutamine' neurodegenerative disorders. This observation strongly suggests that the striatal cell specificity of the polyglutamine expansion in the context of huntingtin must be determined by some aspect of huntingtin's structure, subcellular location or activities [2].

Huntingtin is postulated to function as a flexible ~350 kDa HEAT domain scaffold that may facilitate the assembly and possibly the subcellular location of large protein complexes [3-7]. Huntingtin's large number of diverse cytoplasmic and nuclear protein binding partners strongly suggest that huntingtin may participate in a variety of cellular processes that range from trafficking of growth factor complexes to gene transcription (reviewed in [5,8,9]. However, despite the potential importance of huntingtin's normal function to our understanding of how the dominant polyglutamine mutation causes HD pathology, huntingtin's precise molecular and cellular activities have not been defined.

Therefore, we, and others, set out to discover huntingtin's essential activities by studying the effects of huntingtin deficiency in the mouse. Inactivation of the mouse HD gene (*Hdh*) has shown that huntingtin is not required for cell viability, as evidenced by the survival of mouse embryonic stem cells and neurons that lack huntingtin [10-12]. However, huntingtin is needed at the level of the organism for proper mammalian embryonic development [10,13,14]. Complete lack of huntingtin results in developmental arrest during gastrulation, while severe reduction of huntingtin levels results in abnormal neurogenesis and perinatal lethality [15].

Analysis of huntingtin deficient *Hdh*^*ex*4/5^/*Hdh*^*ex*4/5 ^embryos reveals that homozygous inactivation of the mouse *HD *gene does not overtly affect development until E7.0. By E7.5, mutant embryos exhibit a shortened primitive streak, reduced size and, by morphology, lack a node and head folds. Mutants are rapidly resorbed by E8.0 [10]. Importantly, the expression of huntingtin only in extraembryonic tissues in chimeras rescues this gastrulation phenotype, suggesting that huntingtin is required only in cells of the extraembryonic lineage and acts in a cell non-autonomous manner at this stage [16].

Extraembryonic tissues are essential for supplying nutrients and signals that direct anterior/posterior axis formation and patterning in the developing embryo (reviewed in [17]), implicating huntingtin in either or both of these processes. Of these possibilities, the nutritive role has been more extensively investigated. However, huntingtin deficient embryos do not display obvious visceral endoderm defects, with the notable exception of compromised iron transport in later stage mutants, although iron uptake is undisturbed [16] and endocytosis is not impaired in huntingtin deficient embryos or embryonic stem cells [16,18].

By the same token, huntingtin shuttles through the nucleus, where it is required for proper nuclear localization of its transcription factor partners, suggesting that huntingtin may play a role in transcription cascades in extraembryonic tissues that pattern the embryo [18]. Therefore, we have examined this hypothesis, by monitoring the expression of genes that determine normal embryonic patterning and morphogenesis in *Hdh*^*ex*4/5^/*Hdh*^*ex*4/5 ^huntingtin deficient embryos. Our results support and refine the hypothesis, indicating that huntingtin is required for proper mesoderm patterning and for normal regional restriction of the expression of a subset of growth and transcription factors.

## Results

### Huntingtin-deficient embryos exhibit abnormal streak progression and paraxial mesoderm production

Since extraembryonic tissues supply nutrients to the developing embryo, we tested the possibility that huntingtin deficiency may perturb this function by performing RT-PCR analysis to examine the expression of a panel of 'nutritive' genes in E7.5 wild-type and *Hdh*^*ex*4/5^/*Hdh*^*ex*4/5 ^huntingtin deficient embryos. Consistent with a previous report [16], no obvious differences were found in the expression of "nutritive" genes (*Hnf4*, *Afp*, *Tfn*, *ApoAI*, *Apo-AIV*, and *ApoB*) or genes involved in yolk sac hematopoiesis or vasculogenesis (*Ttr*, *Rbp*, *Flt1*, *Flk1*, *Tal1*, *Rbtn2*, *GATA1*) (data not shown), suggesting that huntingtin is not essential for the proper expression of genes required for the nutritive function of the extraembryonic tissues.

To investigate huntingtin's developmental activities, we then analyzed the expression of genes which pattern the early embryo or mark morphogenic landmarks in wild-type and *Hdh*^*ex*4/5^/*Hdh*^*ex*4/5 ^embryos by whole mount and section *in situ *hybridization. The dissections confirmed previous morphologic data at E7.0–7.5 that all *Hdh*^*ex*4/5^/*Hdh*^*ex*4/5 ^homozygotes exhibit abnormal morphology, including shortened primitive streak and a lack of morphological head folds or node [10,13]. The results of *in situ *hybridization analysis also confirmed that all three germ layers and extraembryonic tissue are formed in huntingtin deficient embryos.

**Figure 1:**
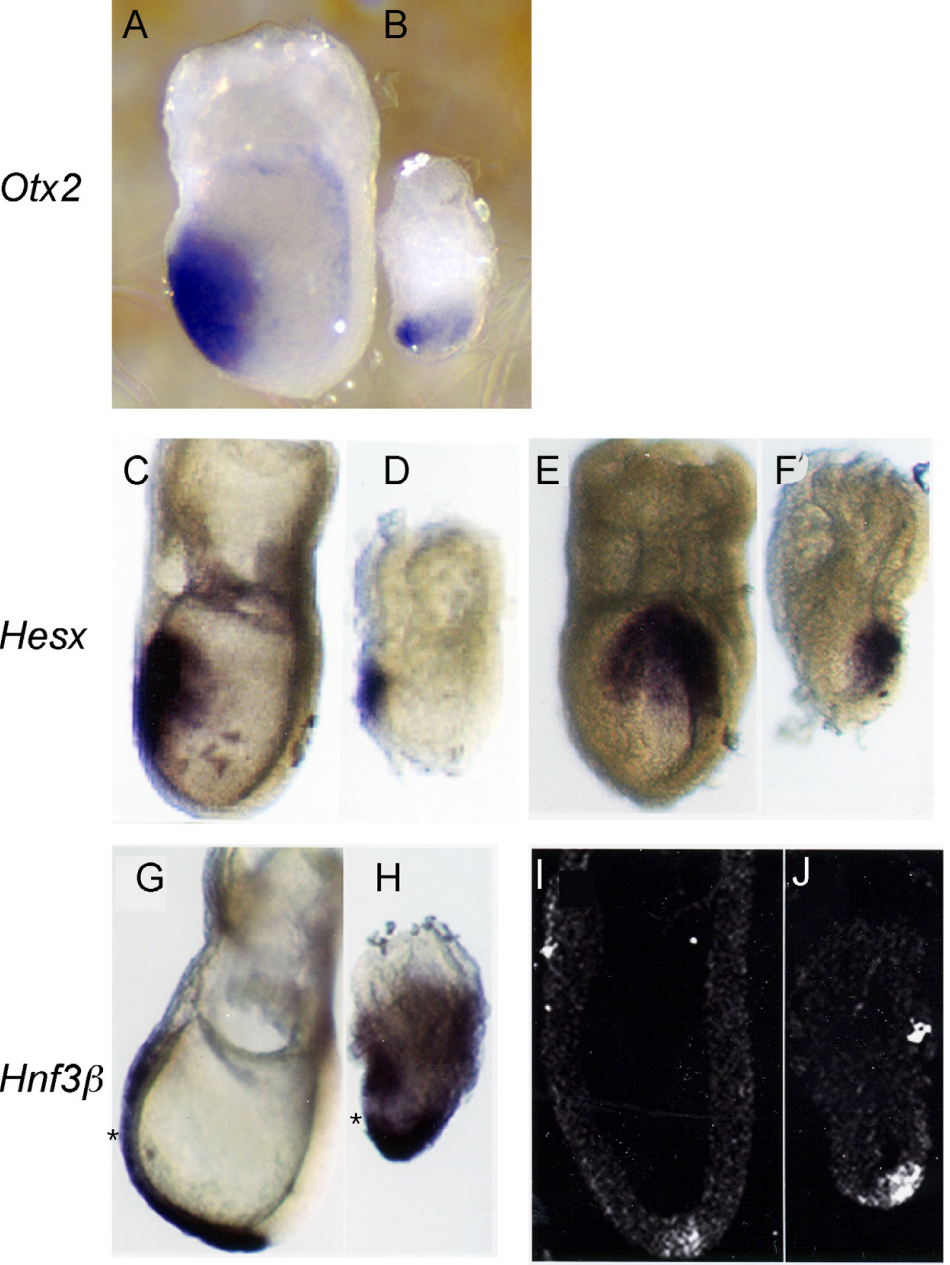
**AVE displacement and anterior neurectoderm induction occur normally in the absence of huntingtin**. Whole mount in situ hybridization analysis of *Otx2 *(A,B) and *Hesx *(C-F) in E7.5 normal (A,C,E) and mutant (B,D,F) embryos reveals that neuroectoderm and anterior visceral endoderm (AVE) develop normally in huntingtin deficient embryos, although the neuroectoderm expression domain is reduced. Asymmetrical expression of *Hesx *in mutant embryos (F) suggests that left-right transcriptional control is maintained. *Hnf3β *expression in the definitive endoderm extends around the distal tip and is reduced in the AVE (*) in both normal (G,I) and mutant embryos (H,J). Taken together, these results suggest normal ectoderm and endoderm induction and localization in *Hdh*^*ex*4/5^/*Hdh*^*ex*4/5 ^embryos. Embryos are shown in lateral views, with anterior to the left in all pictures with the exception of E and F. Embryos are viewed from the anterior aspect in E and F.

*Otx2*, normally expressed in the anterior neuroectoderm and anterior visceral endoderm [19], is expressed in mutant embryos at E7.5 (Fig. 1A,B) although the expression domain appears reduced. Similarly, *Hesx1 *expression is grossly normal in mutant embryos, with expression localized to the AVE and neuroectoderm (Fig. 1C–F, [20]), although the expression domain also appears reduced. These results indicate appropriate specification and movement of anterior visceral endoderm (AVE) cells from the distal tip and suggest that neuroectoderm is induced in the mutant embryos.

To examine definitive endoderm formation, the expression of *Hnf3β *(*FoxA2*) in mutant and wild-type embryos was analyzed. In wild-type embryos, *Hnf3β *expression is confined to the node and anterior definitive endoderm (Fig. 1G,I[21]). Mutant embryos exhibit *Hnf3β*-reactive definitive endoderm over the disorganized anterior streak region and proceeding rostrally around the distal tip (Fig. 1H,J). In both normal and mutant embryos, the AVE exhibits little *Hnf3β *expression. Therefore, huntingtin deficiency does not greatly affect *Hnf3β *regulation or the reorganization of the visceral endoderm.

**Figure 2:**
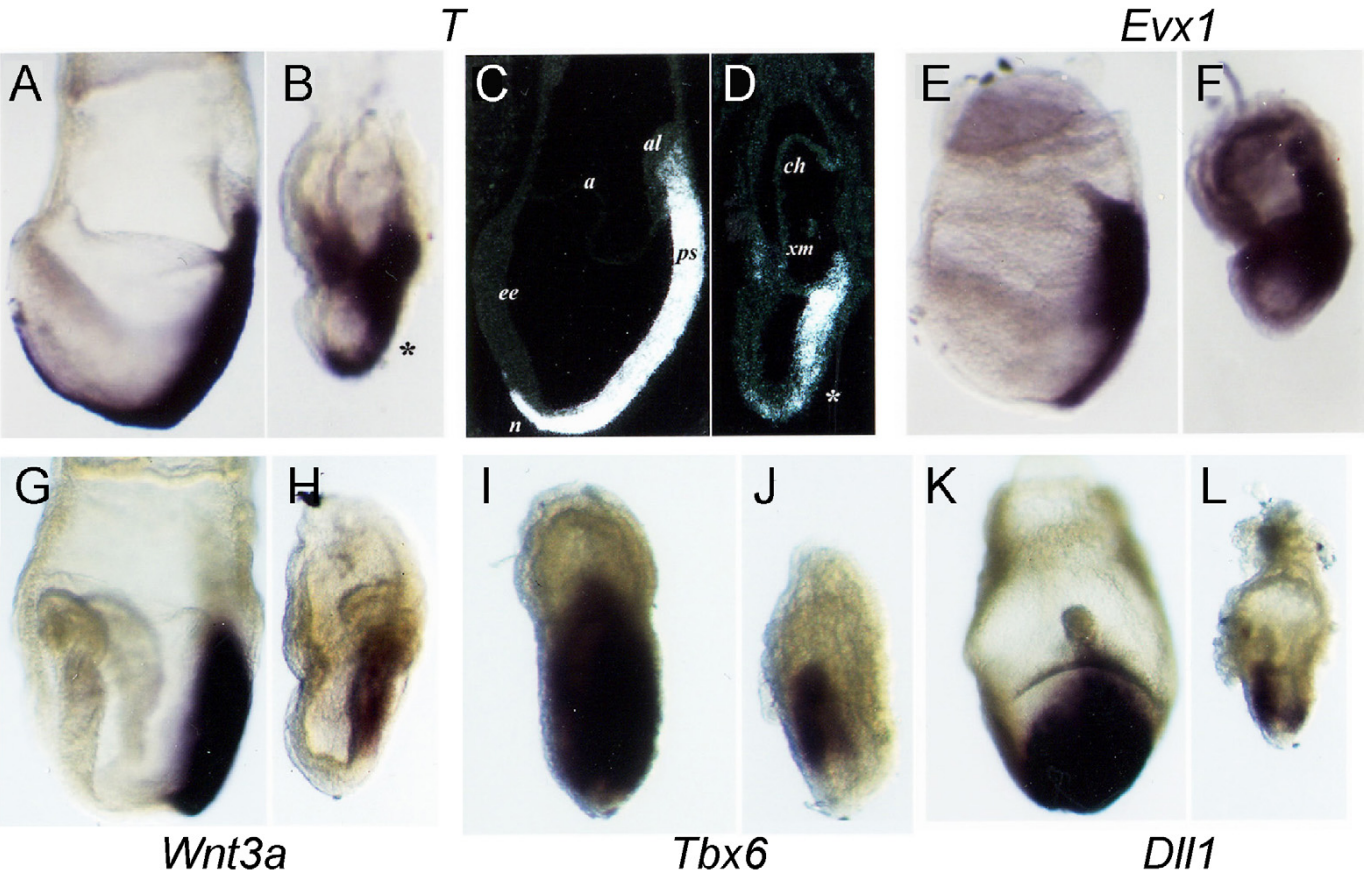
**Huntingtin is required for formation of anterior primitive streak and paraxial mesoderm**. Whole mount and section *insitu *hybridizations of E7.5 embryos shows *T *(*Brachyury*) (A-D) is expressed in the primitive streak, node, axial mesoderm and *Evx1 *(E-F) is expressed in the primitive streak, most strongly in the proximal streak wild-type embryos. However, in mutant embryos, both *T *(B, D) and *Evx1 *(F) are ectopically expressed in the extraembryonic region. *Wnt3A *expression is reduced in mutant embryos (H), although the localization of its expression to the proximal streak is the same as in wild-type embryos (G). Analysis of paraxial mesoderm markers *Tbx6 *(I,J) and *Dll1 *(K,L), reveals that these markers are reduced in mutant embryos (J,L), suggesting impaired paraxial mesoderm production in the absence of huntingtin. Embryos in A-H are shown in a lateral view with anterior oriented to the left. Embryos in I-L are shown in a posterior view (I,K) or near posterior (J,L) view with proximal oriented toward the top. In (C,D), al = allantois, a = amnion, ch = chorion, ee = embryonic node(N), em = extraembryonic mesoderm, n = node, ps = primitive streak. Rather than a node, mutant embryos exhibit a region of disorganized cells (*) at the distal extent of the short primitive streak.

The lack of a morphological node and presence of a shortened streak, together with reduced neuroectoderm and lack of headfolds, suggest that anterior streak formation may be impaired in huntingtin deficient embryos. To investigate this possibility, we examined mesoderm formation in mutant embryos. Mesoderm is specified in the mutant embryos, as marked by the expression of *T *(*Brachyury*) and *Evx1 *(Fig. 2A–F). However, close inspection of the data reveals abnormal patterning within this tissue and its derivatives.*T*, normally expressed in the primitive streak, node and axial head process/notochord mesoderm [22], is detected in the shortened streak and axial mesoderm in *Hdh*^*ex*4/5^/*Hdh*^*ex*4/5 ^embryos, extending rostrally from a region of weakly positive cells (Fig. 2A,B). *T *expression appears weaker, however, in the anterior streak, corresponding to cells that will give rise to axial mesoderm (Fig. 2D). *T *is also ectopically expressed in mutant extraembryonic mesoderm at the anterior embryonic junction and along the chorion (Fig. 2B,D). Similarly, *Evx1*, normally expressed in primitive streak mesoderm at E7.5 with highest levels in proximal cells [23], is expressed in the proximal shortened streak but is also aberrantly expressed throughout the extraembryonic mesoderm, allantois and chorion (Fig. 2E,F). Extraembryonic mesoderm, derived from the proximal streak, does not normally express *T *or *Evx1 *in wild-type embryos [22]. Therefore, the inappropriate expression of *T *and *Evx1*, the shortened primitive streak, and the absence of a morphological node, all suggest that the anterior primitive streak is deficient in the mutant embryos.

The anterior streak generates paraxial mesoderm. Therefore we examined paraxial mesoderm formation in wild-type and mutant embryos, revealing deficits in mesoderm patterning. Starting at E.7.5, *Wnt3A *is expressed in the primitive streak in cells fated to become paraxial mesoderm. In huntingtin deficient mutants, *Wnt3a *is induced in the proximal streak (Fig. 2G,H), confirming stage appropriate posterior development, in contrast to the absence of anterior head folds. However, expression of *Wnt3a *is noticeably reduced in *Hdh*^*ex*4/5^/*Hdh*^*ex*4/5 ^embryos, suggesting a defect in paraxial mesoderm development (Fig. 2H). Reduced expression of *Tbx6 *in the mesoderm lateral to the primitive streak in mutant embryos confirms this interpretation (Fig. 2I,J). Furthermore, in mutant embryos at E7.5, the expression of *Dll1 *in the distal streak region and in only a narrow swath of cells located laterally confirms the paucity of paraxial mesoderm (Fig. 2K,L, [24]). These results strongly suggest that anterior primitive streak formation is impaired, resulting in reduced axial and paraxial mesoderm formation and impaired neural development.

### Impaired regional restriction of growth factor expression in the absence of huntingtin

**Figure 3:**
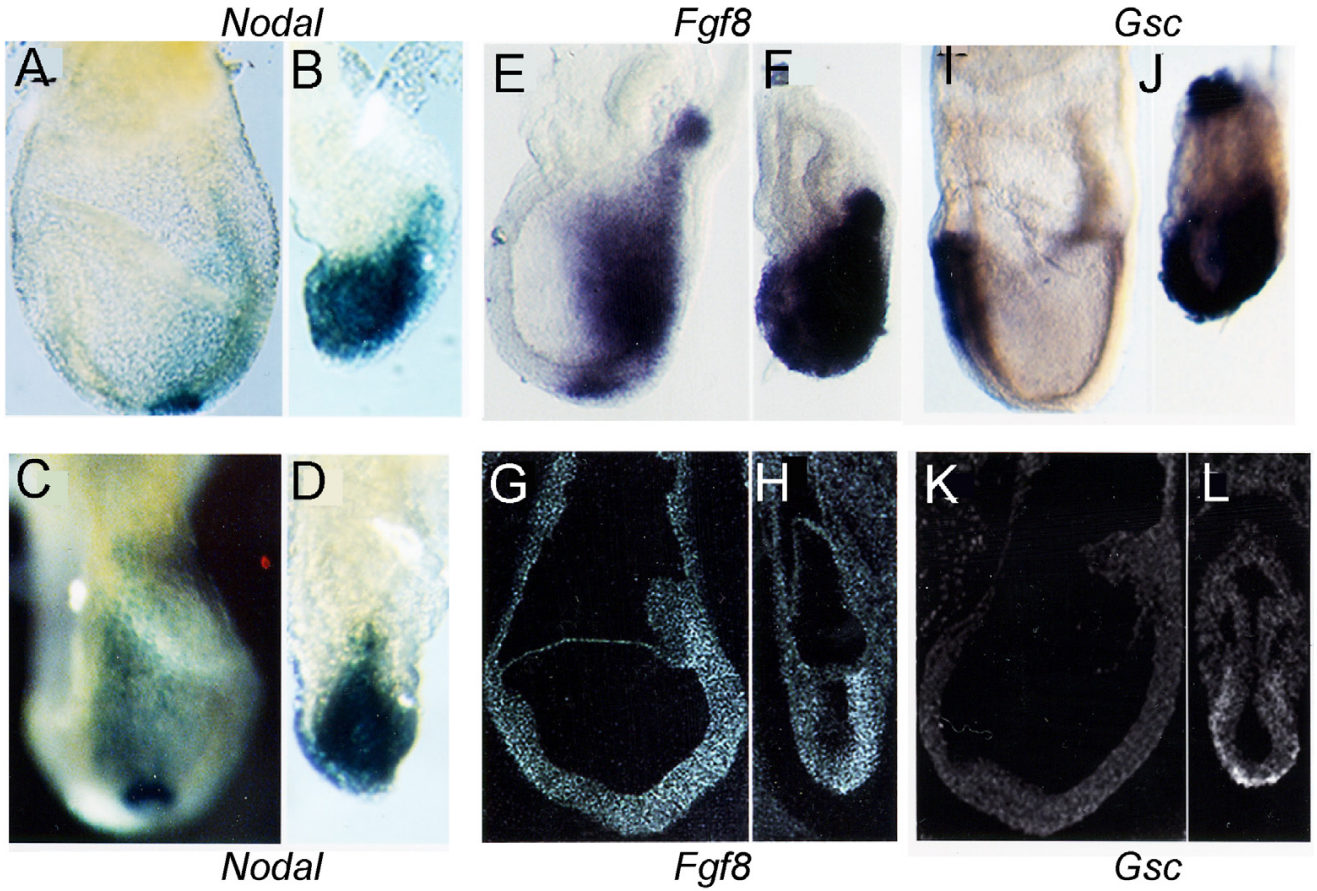
**Impaired regional restriction of gene expression in huntingtin deficient embryos**. X-gal staining of Nodal-LacZ embryos shows staining in endoderm near the node of normal embryos (A,C) but broad staining in mutant embryos (B, D), although expression is higher in the posterior. The tight node expression of *Nodal *in normal embryos (C) is lost in mutant embryos (D), consistent with the loss of a morphological node in the absence of huntingtin. Whole mount and in situ hybridization of E7.5 day embryos reveals that *Fgf8 *is detected in the proximal streak and is downregulated in cells migrating out of the streak in normal embryos (E,G). In contrast, *Fgf8 *remains highly expressed in mutant embryos (F,H). Transient expression of *Gsc *in the definitive endoderm overlying the prospective head region in normal embryos (I,K) is distinguished in other cell layers in normal embryos but remains unrestricted in mutant embryos (J,L). Earlier posterior expression of *Gsc *is also maintained in mutant embryos (J) while it is down-regulated in normal embryos (I). Embryos (A,B,E,F,G,H,I,K,L) are shown in a lateral view with anterior oriented to the left. Embryos (C,D) are in a posterior view.

To elucidate the apparent patterning deficits, we next analyzed signaling molecules that are required for early patterning. *Nodal*, a member of the *Tgfβ *family of secreted molecules is required for the formation and maintenance of the primitive streak and induction of the AVE [25-27]. *Nodal *is normally expressed throughout the epiblast and overlying visceral endoderm at early post implantation stages [28], but later becomes restricted to the posterior of the embryo to the site of primitive streak with asymmetrical visceral endoderm expression marking the left-right axis. By E7.5, *Nodal *expression is restricted to the node. *Nodal *expression was assessed in *Hdh*^*ex*4/5^/*Hdh*^*ex*4/5 ^embryos heterozygous for the *Ndl lac Z *allele [28,29]. Notably, heterozygous loss of nodal does not alter the *Hdh*^*ex*4/5^/*Hdh*^*ex*4/5 ^phenotype, as determined by morphology of *Hdh*^*ex*4/5^/*Hdh*^*ex*4/5^:*Ndllacz*/*Ndl*+ embryos compared with *Hdh*^*ex*4/5^/*Hdh*^*ex*4/5 ^embryos (data not shown). In contrast to wild-type embryos, which exhibit tight restriction of *Nodal.LacZ *expression to the node, *Hdh*^*ex*4/5^/*Hdh*^*ex*4/5^:*Ndllacz*/*Ndl*+ embryos express *Nodal.LacZ *throughout the endoderm overlying the epiblast, with higher levels in the posterior in an asymmetric pattern (Fig. 3A–D). The lack of tight restriction of nodal signal is consistent with a failure to form an organized node structure.

*Fgf8 *signaling is also essential for normal gastrulation in the mouse embryo. *Fgf8 *is required for cell migration away from the primitive streak [30]. Expressed just prior to streak formation in the posterior epiblast and visceral endoderm, *Fgf8 *is restricted to the streak mesoderm at E7.5 in a decreasing proximal-distal gradient and is downregulated in cells shortly after they exit the streak (Fig. 3E,G). In *Hdh*^*ex*4/5^/*Hdh*^*ex*4/5 ^embryos, *Fgf8 *expression is strongly expressed in the posterior region in the primitive streak and ectopically in the endoderm overlying the entire epiblast (Fig. 3F,H). However, streak derivatives appear to migrate normally as evidenced by the proper anterior expression of markers such as *Otx2*, *Hnf3β *and *Hesx1 *anteriorly (Fig. 1). Therefore, mutant embryos exhibit normal migration of streak derivatives but display impaired *Fgf8 *repression in mutant endoderm.

*Hdh*^*ex*4/5^/*Hdh*^*ex*4/5 ^embryos also fail to restrict the expression of *goosecoid *(*Gsc*). Normally, *Gsc *is initially expressed in the visceral endoderm and proximal, posterior streak where the primitive streak will form prior to gastrulation. As the primitive streak forms and extends, *Gsc *is expressed in the distal streak, the node, and the axial mesoderm extending anteriorly from the node (Fig. 3I,K, [31,32]). However, in the mutant *Hdh*^*ex*4/5^/*Hdh*^*ex*4/5 ^embryos, high levels of *Gsc *expression remain unrestricted in the endoderm overlying the entire embryo and ectopically in cells adjacent to the ectoplacental cone (Fig. 3J,L). These results suggest that, in contrast to proper *Hnf3β *regulation, *Gsc *remains inappropriately activated in mutant visceral and definitive endoderm, implicating huntingtin in the proper restriction of this homeodomain transcription factor.

### Huntingtin is not required for expression of extraembryonic signaling molecules

**Figure 4:**
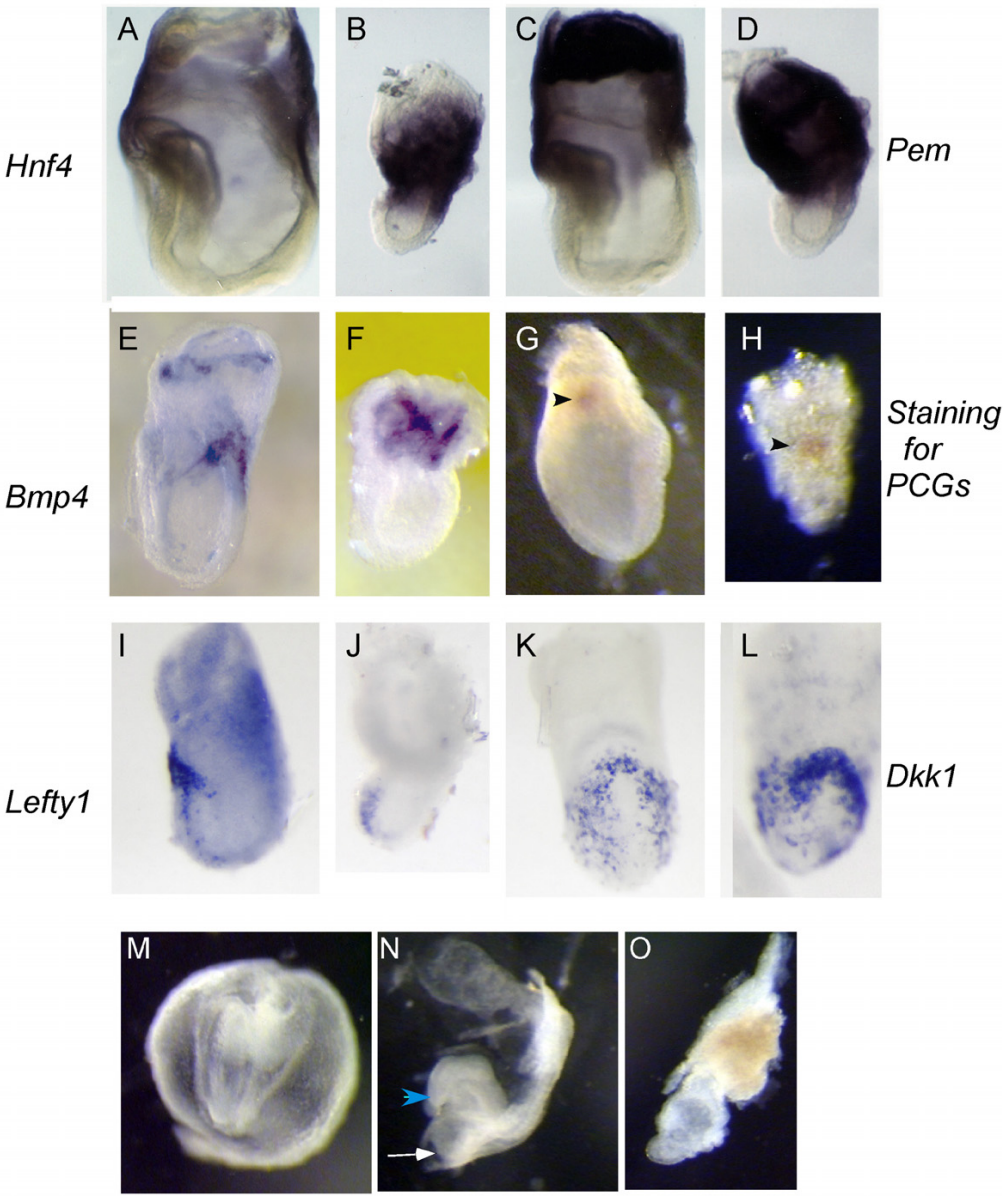
**Normal expression of extraembryonic markers in huntingtin deficient embryos**. Whole mount in situ hybridization analysis at E7.5 of markers of the extraembryonic tissues reveals grossly normal expression in the absence of huntingtin. *Hnf4*, expressed in the visceral endoderm at the junction of embryonic-ectoderm junction (A), is normal in mutant embryos, although the signal is slightly higher (B). Similarly, the expression of *Pem *transcripts is maintained in mutant embryos (D) similar to normal embryos (C), although *Pem *is expressed in the abnormal lopsided overhang of visceral endoderm over the anterior of the mutant embryos. Expression of extraembryonic signaling molecules is unaffected by the loss of huntingtin, as evidenced by the expression of *Bmp4 *(E,F) in the extraembryonic ectoderm, and *Lefty1 *and *Dkk1 *(I-L) in the AVE in mutant embryos. *Bmp4 *is not localized, however, to a ring of extraembryonic ectoderm in mutant embryos (F) as in normal embryos (E). Primitive germ cells (PCGs) are induced normally in both wild-type (G) and mutant embryos (H), suggesting the Bmp4 signaling from the extraembryonic ectoderm to the epiblast is normal. *Lefty1 *expression appears disorganized in mutant embryos (I) compared to wild-type embryos (J). In contrast, the anterior expression of *Dkk1 *in the AVE in mutant embryos (L) matches the wild-type expression pattern (K). Despite normal AVE formation, head folds fail to form in mutant embryos, even when cultured in nutrient rich media for 24 hours. Wild-type E7.5 embryos, when cultured in 75% rat serum, develop somites (M), heart (white arrow, N) and head folds (blue arrow head, N) in culture. In contrast, huntingtin deficient embryos continue to live in culture but do not form headfolds, heart or somites (O). Embryos are shown in a lateral view (A-F, I-J) with anterior oriented to the left. Embryos in (G,H,K,L) are shown in an anterior view with proximal oriented up.

Previous studies of chimeric embryos suggest that huntingtin is required only in the extraembryonic tissue for proper development [16]. Signals from the extraembryonic tissue are critical for the induction of embryonic signals and for patterning the epiblast. Consequently, we examined extraembryonic development in huntingtin deficient embryos. *Hnf4 *is a transcription factor expressed in the primitive endoderm as soon as this tissue becomes distinct and is a key regulator of visceral endoderm secreted factors such as alphafetoprotein, apolipoproteins, and transferrin. Inactivation of *Hnf4 *results in impaired gastrulation [33,34]. At E7.5, *Hnf4 *is expressed in the columnar visceral endoderm cells at the extraembryonic-ectoderm junction (Fig. 4A, [33]). In *Hdh*^*ex*4/5^/*Hdh*^*ex*4/5 ^embryos, consistent with normal primitive and visceral endoderm differentiation, *Hnf4 *expression appears normal, although the signal is stronger in mutant embryos compared to wild-type embryos (Fig. 4B). Similarly, *Pem*, a transcription factor expressed in proximal visceral endoderm and ectoplacental cone in wild-type embryos at E7.5, also is expressed in these tissues in the mutant embryos (Fig. 4C,D[35]). However, *Pem *expressing visceral endoderm hangs over the anterior of the mutant embryos, revealing abnormal location despite grossly normal differentiation.

Signals from the extraembryonic tissues, including the anterior visceral endoderm and extraembryonic ectoderm are required for proper formation and patterning of the epiblast [17]. Bmp4 is a signaling molecule that is first expressed uniformly throughout the extraembryonic ectoderm and subsequently is localized to a ring of extraembryonic ectoderm adjacent to the epiblast (Fig. 4E, [36]). A key factor in regulating the formation of the node and primitive streak, Bmp4 is required for patterning the embryo along the proximodistal axis [37-40]. In the absence of huntingtin, *Bmp4 *expression is properly maintained in the *Hdh*^*ex*4/5^/*Hdh*^*ex*4/5 ^extraembryonic ectoderm but is also expressed throughout the extraembryonic ectoderm (Fig. 4F) in a pattern that is similar to early *Bmp4 *expression rather than being restricted to a ring of extraembryonic ectoderm as seen in the wild-type embryos To assess Bmp4 signaling from the extraembryonic ectoderm, we evaluated primordial germ cells (PGCs), which require Bmp4 for their induction [37]. PGCs can first be detected at E7.0 and subsequently underlie the posterior portion of the primitive streak. Whole mount staining of E7.5 mutant and wild-type embryos for alkaline phosphatase activity reveals that PCGs form in *Hdh*^*ex*4/5^/*Hdh*^*ex*4/5 ^embryos, suggesting that Bmp4 signaling is functional in the absence of huntingtin (Fig. 4G,H).

The anterior visceral endoderm (AVE) is also an extraembryonic source of signals that are critical for early patterning. Wnt and nodal antagonists, *Dkk1 *(*mdkk-1*) and *Lefty1 *respectively, are expressed in the AVE and are important in limiting the posteriorization of the anterior embryo by restricting Nodal and Wnt signaling [41-43]. In *Hdh*^*ex*4/5^/*Hdh*^*ex*4/5 ^embryos, both *Dkk1 *(Fig. 4I,J) and *Lefty1 *(Fig. 4K,L) are expressed normally in the AVE as compared with wild-type embryos. However, *Dkk-1 *levels appear to be slightly increased in *Hdh*^*ex*4/5^/*Hdh*^*ex*4/5 ^embryos, although the pattern of *Dkk-1 *expression remains unchanged and this increase may just reflect the same amount of expression in a smaller area. Therefore, the ectopic expression of *Nodal *(Fig. 3A–D) and the decreased *Wnt3a *expression (Fig. 2H) in mutant embryos do not appear to be result of changes in the expression pattern of *Lefty1 *or *Dkk1*.

Despite normal AVE formation and neuroectoderm induction, head folds do not form in *Hdh*^*ex*4/5^/*Hdh*^*ex*4/5 ^embryos. Therefore, to determine whether mutant embryos are inherently capable of forming head folds, embryos harvested at stage E7.5 were allowed to progress in rich culture medium *in vitro *for 24 hours. Wild-type embryos continued to develop head folds, somites and hearts (Fig. 4M,N). In contrast, mutant stage 7.5 embryos did not develop headfolds, hearts or somites, although these embryos continued to live (Fig. 4O). These results strongly suggest that in the absence of huntingtin, embryos are unable to undergo organogenesis, even if they continue to live past E7.5 in a nutrient rich environment.

## Discussion

We have investigated the embryonic processes that require huntingtin in order to more precisely delineate huntingtin's essential molecular and cellular activities and to provide clues to the mechanism by which the dominant polyglutamine expansion mutation in huntingtin leads to HD pathogenesis. In pursuing the finding that huntingtin is needed only in extraembryonic tissues for normal gastrulation, our data fail to provide evidence of abnormal nutritive gene expression in *Hdh*^*ex*4/5^/*Hdh*^*ex*4/5 ^embryos. Instead, our results reveal that huntingtin is required for normal anterior streak formation and the consequent production of paraxial mesoderm, with a previously unrecognized role for huntingtin in the proper extinction of transiently and/or dynamically expressed genes.

Indeed, the hallmark of the huntingtin deficient molecular phenotype is the impaired down-regulation of a subset of dynamically expressed genes, after the proper onset of expression. This phenomenon does not reflect a lack of anterior/posterior axis formation, as evidenced by the formation of the AVE anteriorly and the primitive streak posteriorly. Nor can it be simply explained by delayed development, as stage-specific markers, such as *Wnt3a *and primordial germ cells, which are detectable at E7.0 in wild-type embryos, are induced appropriately. Furthermore, the expression of *T *and *Evx1 *in the extraembryonic mesoderm of mutant embryos is not a feature of wild-type embryos, even at earlier stages. This strongly suggests that in huntingtin deficient embryos, the migration of the distal streak derivatives to the extraembryonic mesoderm occurs normally but that the down-regulation of these genes is impaired. This impairment may also explain the failure of huntingtin deficient embryos to properly restrict the expression of *Fgf8*, *Nodal *and *Gsc*. Thus, huntingtin may play a direct role in the transcriptional regulation, or mRNA stability of these genes or it may act indirectly by intersecting with other pathways that regulate the expression of these genes.

The requirement for huntingtin in the extraembryonic tissues had prompted us to test whether impaired extraembryonic signals might be responsible for the dysregulation of gene expression within the epiblast that is observed in *Hdh*^*ex*4/5^/*Hdh*^*ex*4/5 ^embryos. Extraembryonic development in *Hdh*^*ex*4/5^/*Hdh*^*ex*4/5 ^embryos is associated with mildly elevated levels expression of *Hnf4 *in the primitive endoderm and *Pem *in the lopsided anterior chorion but the expression of other known signals, such as *Bmp4 *from the extraembryonic ectoderm, and *Dkk1 *and *Lefty1 *from the AVE, appear to be normal, although the slight increase in *Dkk-1 *expression in *Hdh*^*ex*4/5^/*Hdh*^*ex*4/5 ^embryos suggests that further investigation into Wnt signaling is warranted. Moreover, extraembryonic Bmp4 signaling is not impaired in the absence of huntingtin, as the induction of PCGs in mutant embryos is normal, implying proper transport and secretion of the appropriate extraembryonic signals. However, *Nodal*, *Fgf8 *and *Gsc *are expressed ectopically in the visceral endoderm of *Hdh*^*ex*4/5^/*Hdh*^*ex*4/5 ^embryos. Both Nodal and Fgf8, important growth factors required for normal development of the epiblast, are tightly regulated during gastrulation. Therefore, misexpression of either or both of these factors, or of goosecoid, in the visceral endoderm could contribute to the *Hdh*^*ex*4/5^/*Hdh*^*ex*4/5 ^mutant phenotype. In addition, it is possible that other extraembryonic signal(s) that we have not analyzed may also be affected by the lack of huntingtin activity in extraembryonic cells in mutant embryos.

Huntingtin deficient embryos also fail to form headfolds, and to undergo organogenesis, even after culturing in nutrient rich media. The absence of headfold formation in these embryos does not appear to be a result of a failure to induce neurectoderm or a failure to form the AVE, since mutant embryos express markers such as *Otx2*, *Ddk1*, *Lefty1 *and *Hesx1*. In addition, since node formation is not required for neural induction [44-46], the failure to form a node in huntingtin deficient embryos is also unlikely to explain the lack of headfolds. The apparent reduction of paraxial mesoderm in *Hdh*^*ex*4/5^/*Hdh*^*ex*4/5 ^embryos could explain the lack of headfolds since paraxial mesoderm is important for the full development of neuroectoderm, and consequently, headfolds. Alternatively, the inability to manifest headfolds could suggest that huntingtin is required at a very early stage for normal CNS development. This conclusion is consistent with the finding that severely reduced levels of huntingtin, from a hypomorphic *Hdh *allele, lead to abnormal brains later in embryonic development [15].

The cardinal features of complete *Hdh *inactivation that we observe are similar to the phenotypes that stem from the complete inactivation of the Polycomb group gene (Pc-g) *Eed *(embryonic ectoderm development). Indeed, complete deficiency for either huntingtin or the eed protein leads to abnormal streak development, lack of headfold formation, ectopic *T*, *Evx1 *and *Nodal *expression and disruption of anterior primitive streak mesoderm production [47]. Interestingly, Eed protein is also required for proper trophoblast development and normal maintenance of imprinted X-inactivation and genomic imprinting [47-49], suggesting that these activities warrant investigation in huntingtin deficient embryos.

Thus, our observations provide unexpected starting-points in the search for huntingtin's precise molecular activities, which began with the discovery that this HEAT domain protein hosts the dominant polyglutamine property that is the fundamental basis of HD pathogenesis. In HD patients and in accurate genetic replicas, *HD *CAG knock-in mice, the dominant mutation specifically affects the major population of neurons in the striatum, without impairing huntingtin's essential activities in embryonic development [50-53]. Indeed, homozygous HD patients make no wild-type huntingtin, and, in the mouse, a single mutant *Hdh *allele's worth of mutant huntingtin can fully rescue huntingtin deficiency embryonic phenotypes [15,51]. The quest to understand the HD mechanism, therefore, is aimed at delineating the huntingtin activity that may explain the striatal cell specificity of the polyglutamine mutant version of huntingtin. One hypothesis is that huntingtin is normally involved in gene transcription, as proposed for NRSF/REST mediated BDNF expression [54]. Now, our finding that huntingtin can be absolutely necessary for the appropriate regulation of genes with dynamic expression patterns *in vivo*, provides a compelling reason to elucidate the cellular machinery that is necessary for huntingtin mediated gene regulation.

## Conclusion

Our findings indicate that huntingtin is required for proper patterning of the epiblast during early embryogenesis, for proper anterior streak and node formation, primitive streak progression, paraxial mesoderm and head fold formation, as well as for the proper restriction of transiently expressed growth and transcription factor genes. Knowledge of the molecular basis of these changes in huntingtin deficient embryos should facilitate the identification of the cellular pathways that are dependent on huntingtin activities. These will be important for implicating candidates to be assessed in the extraembryonic signals that determine anterior streak progression in the developing embryo and in delineating the dominant activity of the polyglutamine tract in huntingtin that determines the striatal specificity of HD.

## Methods

### Mice and genotyping

The *Hdh*^*ex*4/5 ^mice carrying a *pGKneo *insertion/replacement inactivating mutation in the mouse *HD *gene homologue have been described previously [10]. The experiments were conducted in accordance with an IACUC approved protocol, through the MGH Subcommittee on Animal Research. Mutant *Hdh*^*ex*4/5^/*Hdh*^*ex*4/5 ^and normal littermates were obtained in timed pregnancies from mating of *Hdh*^*ex*4/5^/*Hdh*^+ ^heterozygotes, genotyped by PCR assay, as described [10]. The day of plug was taken to be E0.5. Embryos that were morphologically normal were pooled separately from morphologically mutant embryos for analysis. Nodal expression was determined in embryos from matings of *Hdh*^*ex*4/5^/*Hdh*^+^; *Ndll*^*acZ*^/*Ndl*^+ ^compound heterozygotes genotyped by PCR assay as described in [29].

### Whole mount and section *in situ *hybridization and β-gal staining

After dissection in PBS, embryos were fixed overnight in 4% paraformaldehyde at 4°C. For sections, decidua fixed in 4% paraformaldehyde, were embedded in paraffin and sectioned at 7 microns. RNA *in situ *hybridizations were performed as described previously [55]. Nodal.lacZ expression was assessed by β-galactosidase staining as reported [29], on embryos post fixed in 4% paraformaldehyde. Embryos were mounted in 80% glycerol before being photographed.

The huntingtin deficient phenotype is fully penetrant at each of the stages that were assessed [10]. Three to six embryos were evaluated for each marker, with every embryo exhibiting the same mutant phenotype in each case.

### Alkaline phosphatase staining of Primordial Germ Cells (PCGs)

After dissections, embryos were fixed in 4% paraformaldehyde briefly and washed and stored in 1 × PBS/0.1% TX-100 at 4°C. Embryos were washed once with Tris-Maleate Buffer (25 mM Tris-Maleate, pH = 9.0, 0.8 mM MgCl_2_) and were subsequently incubated in alkaline phosphatase staining solution (25 mM Tris-Maleate, pH = 9.0, 0.8 mM MgCl_2_, 0.4 mg/ml alpha-naphthyl phosphate, 1 mg/ml Fast-Red). Stained embryos were washed in 1 × PBS/0.1% TX-100.

### Whole embryo culture

Embryos were dissected at E7.5 and washed in DMEM. Embryos were then cultured individually in 1 ml of culture media (75% immediately centrifuged rat serum and 25% DMEM [56]) for 24 hours while rotating in a 37°C incubator in 5% CO_2_. Embryos were then fixed in 4% paraformaldehyde for analysis.

## Abbreviations

AVE, anterior visceral endoderm; *HD*, Huntington's disease gene; HD, Huntington's disease; *Hdh*, mouse *HD *gene homologue; PCGs, primordial germ cells

## Authors' contributions

JMW, TC, PH-M and MD performed whole mount and *in situ *hybridization assays. MEM and RC contributed to the conception of this study. JMW, TC, PH-M and MEM drafted the manuscript and RC contributed to its finalization. All authors read and approved the final manuscript.
